# Discovery of common marburgvirus protective epitopes in a BALB/c mouse model

**DOI:** 10.1186/1743-422X-6-132

**Published:** 2009-08-27

**Authors:** Warren V Kalina, Kelly L Warfield, Gene G Olinger, Sina Bavari

**Affiliations:** 1 Division of Bacteriology, United States Army Medical Research Institute of Infectious Diseases, Fort Detrick, Fort Detrick, Maryland, 21702, USA; 2 National Biodefense Analysis and Countermeasures Center, Frederick, MD 21702, USA; 3 Integrated Biotherapeutics, Inc., 20358 Seneca Meadows Parkway, Germantown, MD 20876, USA; 4 Division of Virology, United States Army Medical Research Institute of Infectious Diseases, Fort Detrick, Fort Detrick, Maryland, 21702, USA

## Abstract

**Background:**

Marburg virus (MARV) causes acute hemorrhagic fever that is often lethal, and no licensed vaccines are available for preventing this deadly viral infection. The immune mechanisms for protection against MARV are poorly understood, but previous studies suggest that both antibodies and T cells are required. In our study, we infected BALB/c mice with plaque-purified, nonlethal MARV and used overlapping peptides to map H2^*d*^-restricted CD8+ T-cell epitopes.

**Methods:**

Splenocytes from mice infected with nonlethal MARV were harvested and stimulated with multiple overlapping 15-mer peptide pools, and reactive CD8+ T cells were evaluated for antigen specificity by measuring upregulation of CD44 and interferon-γ expression. After confirming positive reactivity to specific 15-mer peptides, we used extrapolated 9-mer epitopes to evaluate the induction of cytotoxic T-cell responses and protection from lethal MARV challenge in BALB/c mice.

**Results:**

We discovered a CD8+ T-cell epitope within both the MARV glycoprotein (GP) and nucleoprotein (NP) that triggered cytotoxic T-cell responses. These responses were also protective when epitope-specific splenocytes were transferred into naïve animals.

**Conclusion:**

Epitope mapping of MARV GP, NP, and VP40 provides the first evidence that specific MARV-epitope induction of cellular immune responses is sufficient to combat infection. Establishment of CD8+ T-cell epitopes that are reactive to MARV proteins provides an important research tool for dissecting the significance of cellular immune responses in BALB/c mice infected with MARV.

## Background

Marburgvirus (MARV), a member of the *Filovirus *family, causes severe hemorrhagic fever concomitant with coagulation anomalies resulting in massive vascular leakage, organ failure, and death in humans and nonhuman primates. MARV is primarily transmitted through contact with infected bodily fluids or tissues of humans or animals, such as bats and nonhuman primates [1]. Other than supportive care, which increases the chance of survival, there is currently no cure for this deadly infection [2,3].

Many reports have characterized filovirus-specific antibody responses in an effort to evaluate the host's overall capacity to fight infection [4-9], and most vaccine studies have relied on antibody titer measurements to predict protection [4,7,10]. MARV-specific, plaque-reducing/neutralizing antibodies alone only partially protect guinea pigs from a MARV infection [11]. In contrast, Ebola virus (EBOV) glycoprotein (GP)-specific monoclonal antibodies can protect infected mice and guinea pigs [6,9], and EBOV-specific antibodies passively transferred into naïve mice result in full protection and a specific de novo cellular response against the virus [9]. However, studies to date have shown that EBOV-neutralizing antibodies are completely ineffective in rhesus macaques [5], which suggests that other immunological mechanisms (i.e., cellular immunity) are needed, either separately or in conjunction with antibodies, for full protection [12].

There is little information available on the induction of cytotoxic T-cell-mediated immunity in response to MARV infection, and the potential role of cytotoxic lymphocytes (CTLs) generated from MARV vaccines has not been investigated. Wang et al. [7] demonstrated that cell-mediated immune responses are generated by an adenovirus-vector MARV vaccine candidate; however, it is not known if such a response is protective or if antibody responses in conjunction with CTLs are needed for complete protection. Several reports have shown that CTLs are the primary protective arm of the acquired immune system involved in fighting off viral infections. Studies involving epitope-specific CTLs against West Nile virus were solely protective when transferred into naïve animals prior to viral challenge [13]. EBOV CTLs specific for an immunodominant T-cell epitope in the viral nucleoprotein (NP) were protective when transferred into naïve BALB/c mice before challenge [14]. EBOV CD8+ T-cell epitopes were mapped in H2^*d*^- and H2^*b*^-restricted cells from BALB/c and C57BL/6 mice and are currently used to determine the presence of CD8+ T-cell responses to EBOV [15]. T-cell-deficient mice vaccinated with Ebola virus-like particles (VLP) succumb to lethal EBOV challenge – a response primarily mediated by CD8+ T cells, with a lesser role for CD4+ T cells [8]. In contrast, adoptive transfer studies of E-specific CTLs from Japanese encephalitis virus do not protect mice without E-specific antibodies [16]. Therefore, depending on the viral infection, antibodies or CTLs alone may be required to eliminate certain viral infections; however, it is likely that MARV protective immunity requires a combination of both.

Based on the previous studies performed with EBOV and the extensive studies carried out on MARV with respect to antibody-mediated protection, it seemed highly likely that cellular immunity contributes to the host's protective immune response against MARV. To determine the importance of T-cell responses during MARV infection, we infected mice with a nonlethal MARV Ravn isolate [17] and approximately 2 weeks later harvested splenocytes from convalescent mice. The identification of CTL epitopes from GP, NP, and VP40 was based on the upregulation of CD44 and interferon-γ (IFNγ) production in CD8+ T cells from this cell population following stimulation with synthetic 15-mer peptides representing the entire translated GP, NP, and VP40 proteins. To explore whether peptide-stimulated MARV convalescent splenocytes could protect naïve mice from MARV challenge, we used a recently developed BALB/c mouse model in which MARV infection causes 100% lethality [17]. We found that several MARV-specific CTL epitopes, which were common to strains Ravn, Ci67, and Musoke, provided significant protection against lethal MARV Ravn challenge in naïve mice. Overall, the discovery of epitope-specific CD8+ T-cell populations that can confer protection against MARV highlights the importance of cell-mediated immunity in the BALB/c mouse model.

## Results

### Preliminary screen of MARV epitopes using overlapping peptide pools

**Figure 1:**
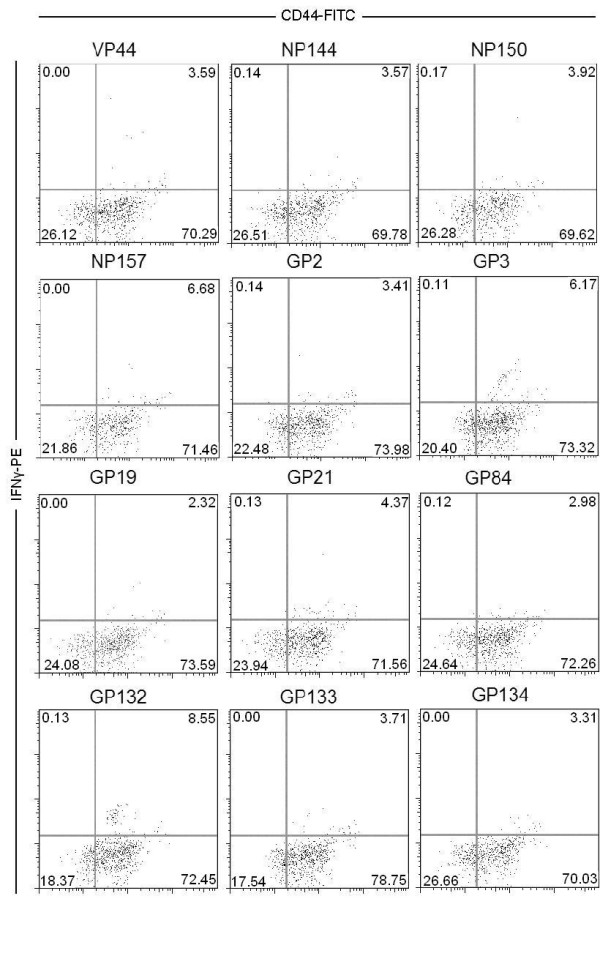
**MARV-specific splenocytes were stimulated with the following 15-mer peptides: VP44, NP144, NP150, NP157, GP2, GP3, GP19, GP21, GP84, GP132, GP133, and GP134**. After stimulation, intracellular levels of IFNγ were measured in gated CD8+ T cells with high CD44 surface expression. Each of the 15-mers induced splenocytes, from previously MARV infected animals, to produce varying amounts of IFNγ. The negative control (irrelevant peptide, EBOV NP12), which was the same in Figure 1 and 2, did not stimulate MARV specific splenocytes to produce IFNγ and the positive control (PMA + ionomycin) did induced IFNγ production.

We used overlapping peptide pools to simplify mapping of reactive 15-mers. Each peptide pool, which contained thirteen 15-mer peptides, contained a new nonoverlapping 15-mer at each increment. Reactivity to two separate peptide pools, which contained only one overlapping duplicate peptide, prompted testing for reactivity to the individual 15-mer peptide. From the initial screen in mice previously infected with the nonlethal, wild-type strain of MARV-Ravn, we found eighteen 15-mer epitopes from GP, six from NP, and two from VP40 that stimulated splenocytes from MARV-infected mice to secrete IFNγ (data not shown). After retesting with individual 15-mer peptides, twelve 15-mer epitopes induced MARV-specific splenocytes to secrete IFNγ at levels greater than 2% above background (see Table 1 and Figure 1). Background, in this case, was determined to be the amount (typically less than 0.5%) of IFNγ secreted from CD8+ T cells after stimulation with an irrelevant EBOV NP peptide. Figure 1 shows data (one of two duplicated samples) from gated CD8+ T cells derived from the spleen of BALB/c mice previously infected with nonlethal MARV. In all cases a small fraction of the activated CD44+ cell population demonstrated secretion of IFNγ after peptide stimulation. Table 1 presents the mean amounts of IFNγ secreted from stimulated CD8+ T-cell populations minus the mean background from CD8+ splenocytes stimulated with an irrelevant peptide from EBOV NP (experiments performed in duplicate).

**Table 1:** Selection of MARV epitopes

Peptide Pool^*a*^	+ Peptide from Pool^*b*^	15-mer Sequence	% CD8 IFNγ^*c*^	9-mer Peptide Motif Results^*d*^	*% CD8 IFNγ*^*c*^
GP1-13	GP2	FLISLILIQGTKNLP	2.85	ILIQGTKNL	2.83
GP1-14	GP3	ILIQGTKNLPILEIA	5.09	QGTKNLPIL	3.46
GP2-17	GP19	TCYNISVTDPSGKSL	2.34	VTDPSGKSL	1.68
GP2-19	GP21	SGKSLLLDPPTNIRD	2.6	LLLDPPTNI	1.72
GP7-17	GP84	SPPPTPSSTAQHLVY	3.11	TPSSTAQHL	2.93
GP11-13	GP132	GILLLLSIAVLIALS	8.36	LLLSIAVLI	8.94
GP11-14	GP133	LSIAVLIALSCICRI	2.96	LSIAVLIAL	1.18
GP11-15	GP134	LIALSCICRIFTKYI	2.73	IALSCICRI	4.12
NP1-20	NP144	AINSGIDLGDLLEGG	2.88	NSGIDLGDL	4.13
NP2-13	NP150	KFNTSPVAKYLRDAG	2.53	NTSPVAKYL	4.71
NP2-20	NP157	EPHYSPLILALKTLE	3.48	HYSPLILAL	2.79
VP6-12	VP44	QHKNPNNGPLLAISG	3.17	KNPNNGPLL	2.38

*Tested in adoptive transfer studies

^a^Pools of peptides were each number represents a new 15-mer peptide

^b^Selected 15-mer peptides capable of restimulating splenocytes of MARV-infected mice

^c^Mean IFNγ levels from two experiments minus mean background

^d^9 mers derived from HLA binding predictions

**Figure 2:**
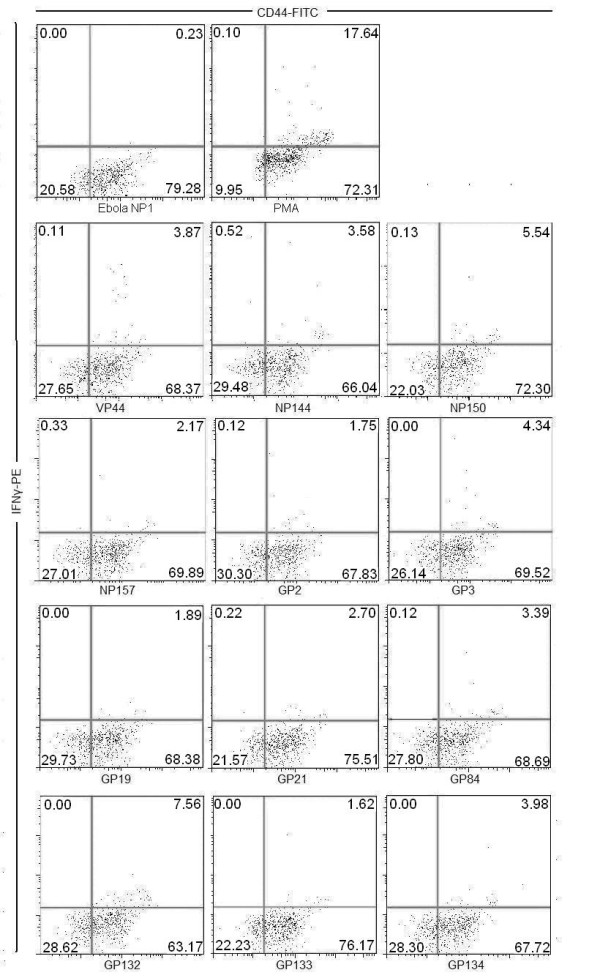
**9-mer peptides, derived from the original 15-mer peptides based on HLA binding predictions, were used to stimulate MARV-specific splenocytes**. IFNγ levels were measured in gated CD8+ T-cell populations with high CD44 surface expression. Each 9-mer stimulated CD8+ T cells to produce varying amounts of IFNγ; whereas, the negative control (EBOV NP12) stimulated splenocytes produced minimal IFNγ.

We then used HLA binding predictions to determine the probable MHC-class I bound 9-mer sequence from each confirmed 15-mer [16]. This program predicted the average half time of disassociation for peptide/MHC class I molecules. Nine-mers, selected by computer predictions for H2^*d*^-restricted mice haplotype, were used to stimulate MARV-specific splenocytes. We found that 9-mer epitopes from GP2, GP3, GP19, GP21, GP84, GP132, GP133, GP134, NP144, NP150, NP157, and VP44 induced IFNγ secretion (greater than 1%) from CD44+ and CD8+ splenocytes. Data from one of two sets of MARV-specific CD8+ splenocytes stimulated with 9-mer peptides is shown in Figure 2. As expected, the 9-mer-stimulated splenocytes demonstrated a similar response, with respect to IFNγ secretion, as the 15-mer-stimulated splenocytes shown in Figure 1.

### MARV 9-mer epitopes induce lytic function

Several of the 9-mer epitopes that produced IFNγ responses in greater than 2% of CD8+ splenocytes were tested for induction of lytic function in CTL responder cells derived from MARV VLP-vaccinated mice. MARV VLP-vaccinated mice were used in this surrogate system under biosafety level 2 conditions because of the lack of appropriate equipment for reading ^51^Cr assays in our biosafety level 4 laboratory. MARV CTL assays were performed with peptide-pulsed PB1 target cells and MARV VLP-vaccinated mice splenocytes as effectors, which were restimulated in the presence of a specific peptide. Spontaneous background (chromium release in assays using nonpulsed target cells) was subtracted from total lysis in each sample being tested. We found that the strongest IFNγ inducing peptide also demonstrated strong T-cell lytic function. Table 2 shows the results of these CTL assays.

**Table 2:** Functional immunological properties from MARV epitopes

Name and Location	9-mer Sequence	% Lysis on CTL Assay^*a*^	Adoptive Transfer % Survival^*b*^
VP44	KNPNNGPLL	6	40
NP144	NSGIDLGDL	16	80
NP150	NTSPVAKYL	15	20
NP157	HYSPLILAL	Not done	10
GP2	ILIQGTKNL	17	50
GP3	QGTKNLPIL	20	20
GP21	LLLDPPTNI	Not done	0
GP84	TPSSTAQHL	Not done	0
GP132	LLLSIAVLI	40	100
GP133	LSIAVLIAL	Not done	20
GP134	IALSCICRI	6	40
PBS	N/A	2	10

^a^% of cells lysed when compared to Triton-X-treated targets

^b^Survival after transfer of epitope-specific splenocytes and challenge with lethal PFU (~1000) of MARV, n = 10 BALB/c mice/group

### MARV 9-mer epitopes protect against MARV challenge

**Figure 3:**
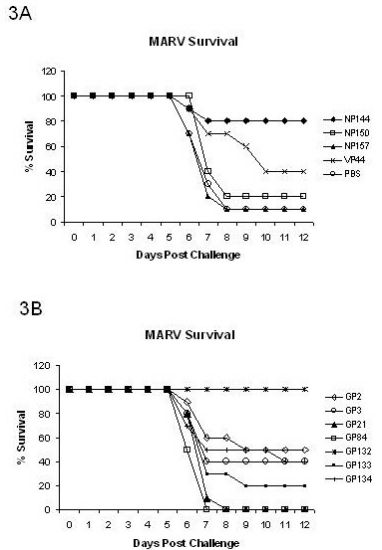
**A, Survival rates for mice receiving NP and VP40 9-mer-stimulated splenocytes prior to lethal MARV challenge**. NP144-stimulated splenocytes offered significant protection (8/10; p < 0.05) against lethal MARV challenge when compared to nonstimulated splenocytes when transferred into naïve mice (1/10). NP150-, NP157-, and VP44-stimulated MARV-specific splenocytes did not significantly protect naïve mice from lethal MARV challenge. B, Splenocytes from previously MARV-infected mice were stimulated with 9-mer peptides and transferred into naïve animals prior to lethal MARV infection. Survival rates were monitored up to 12 days postinfection. Naïve BALB/c mice receiving GP132-stimulated splenocytes were fully protected from lethal MARV (10/10; p < 0.05). GP2-, GP3-, GP21-, GP84-, GP133-, and GP134-stimulated and transferred splenocytes did not individually protect naïve mice from lethal MARV challenge when compared to mice receiving nonstimulated splenocytes from mice previously infected with nonlethal MARV (1/10).

To show that epitope-specific splenocytes could be responsible for an effective T-cell response against MARV in BALB/c mice, we adoptively transferred epitope-specific lymphocytes to naïve mice and challenged them with lethal MARV [17]. Splenocytes were harvested from convalescent mice previously infected with nonlethal MARV, stimulated with GP2, GP3, GP21, GP84, GP132, GP133, GP134, NP144, NP150, NP157, or VP44 for 7 days, and then transferred into naïve mice before being challenged with lethal MARV. We determined that adoptively transferred splenocytes stimulated with the MARV GP132 9 mer completely protected naïve mice from lethal MARV (100% protection, p < 0.05) (Figure 3A). NP144 was significantly protective even though some deaths were recorded (80% protective, p < 0.05) (Figure 3B). Moderate levels of protection were afforded by the T cells stimulated with the GP2 (50%), GP134 (40%), NP150 (20%), and VP44 (30%); however, these levels were not significantly protective when compared to levels in mice given un-stimulated splenocytes (Figure 3). We also tested NP157, GP21, GP84, and GP133 9-mer-stimulated splenocytes in the mouse-MARV adoptive transfer model. We found that none of these offered protection and that the mice demonstrated similar survival rates as those of the control group (see Table 2). In addition, splenocytes stimulated with GP132 or NP144 and then transferred to naïve mice did not protect against mouse adapted EBOV challenge (see Table 3).

**Table 3:** Specificity of MARV epitope specific splenocytes

*Stimulus*^*a*^	*Challenge agent*^*b*^	*% Survival*
NP144	EBOV	10
GP132	EBOV	0
PBS	EBOV	10

^a ^2 mg/ml of peptide was used to stimulate MARV-specific splenocytes

^b^Each mouse was challenged with 1000 PFU of lethal EBOV, n = 10 BALB/c mice/group

## Discussion

Collectively, the data presented here demonstrate that MARV CTL epitopes are present in BALB/c mice and are important during viral elimination. We discovered two CD8+ T cell epitopes for MARV that are conserved among all published strains of the virus. This information can be used for diagnostic assays aimed at determining CD8+ T-cell responses to vaccines or confirmation of a CTL response to infection. In this communication we have shown that both 15-mer and 9-mer GP132 and NP144 peptides stimulated splenocytes as evidenced by upregulation of CD44 expression and secretion of IFNγ (Figure 1 and 2). Nine-mer NP144- and GP132-stimulated splenocytes also demonstrated killing activity by lysing corresponding peptide-pulsed target cells. In addition to cytokine production and lytic activity, NP144- and GP132-stimulated splenocytes protected naïve mice from lethal MARV infection after adoptive transfer (see Table 2 and Figure 3A and 3B).

Overall, GP132-stimulated splenocytes consistently generated higher IFNγ levels and lytic activity than NP144-stimulated cells and, likewise, protected naïve mice from lethal MARV more effectively (see Table 2 and Figure 3A and 3B). Strong lytic function by CTLs is required for EBOV protection [18], and this is likely true for MARV as well. It is worth mentioning that GP134, NP150, and VP44 all had lower lytic function and were likewise less protective; however, NP144 had lower lytic function than GP2 and GP3 but was more protective in the adoptive transfer experiment. Similar to other viral infections, the data support that lytic function is a good indicator of a protective T-cell epitope; however, this may not be the only indicator [19]. Compared to GP132, NP144 is not an immunodominant epitope. Subdominant epitopes to viral proteins may have unpredictable effects on the host response to a virus. A subdominate epitope from the respiratory syncytial virus M2 protein, for instance, still cleared virus and prevented weight loss [20]. From the data presented in this manuscript, it appears that MARV requires immunodominant epitopes for clearance and full protection.

MHC-class I presentation of viral peptides is essential for CD8+ T-cell activation, proliferation, and killing. MHC class I presentation of EBOV and MARV epitopes has not been extensively investigated. It has been shown that blood-derived cells upregulate MHC class II (i.e., HLA-DR) during an EBOV infection, but there are no published reports of MHC class I upregulation or downregulation in blood-derived monocytes, tissue macrophages, or dendritic cells, which are the primary target cells during EBOV or MARV infection [21]. However, Harcourt *et al*. demonstrated that MHC class I is downregulated in EBOV-infected human umbilical vein endothelial cells [22]. In contrast, MARV VLPs upregulate MHC class I on monocytes and dendritic cells, but infection of these cell types with live EBOV or MARV does not produce such an effect (unpublished observation) [23,24]. Incidentally, it also has been shown that overexpression of EBOV GP caused downregulation of MHC class I in 293T cells [25]. There is likely a small amount of antigen processing and presentation on MHC class I before cellular dysfunction; therefore, minuscule amounts of processed antigen on MHC class I, some of which would be the GP132 epitope, may be all that is needed for CD8+ T cell recognition and killing. Viral subversion mechanisms, such as downregulation of MHC class I and co-stimulatory molecules dampen primary immune responses but are less effective during secondary or mature immune responses, such as the case when previously stimulated MARV-specific splenocytes are transferred into naïve animals. In fact, it has been shown that mature CD8+ T cells require far less co-stimulation to kill a specific target [26].

The GP132 epitope is located in the transmembrane portion of the GP(2) domain. In a concurrent GP vaccine study, guinea pigs were vaccinated with a MARV GP(2)-based vaccine and all survived MARV challenge, despite low preinfection antibody titers (data not shown). This suggests that good CTL responses are generated from epitopes contained in the GP(2) portion of the MARV GP. The mucin-like domain of GP has been reported to have toxic effects on cells, and its removal from GP-based vaccines is being explored. There have also been several vaccine strategies that have relied on fusion between the receptor-binding domains (RBD) of GP(1) and GP(2) [27-29]. The majority of 9 mers tested in this study stimulated MARV-specific splenocytes and those shown to protect animals were in the RBD domain, which suggests that this domain is advantageous for antibody and cell-mediated protection. In addition, epitopes GP2, GP3, GP133, GP134 (also located within the RBD domain) stimulated IFNγ production but were not protective. It is possible that these, as well as NP157 and NP150, were not immunodominant and would thus require greater numbers of effector cells to afford protection from a lethal MARV challenge.

Our results showed that MARV epitopes can be good diagnostic indicators of an active cellular immune response to MARV in BALB/c mice. Several concurrent vaccine platforms under investigation most likely rely on CD8+ T-cell-mediated immunity to protect against MARV including the adenovirus-GP [7], the replicon-GP [4], the VSV-GP [30], and VLP-based vaccines [31-33]. Vaccines tested in BALB/c mice can be evaluated by ascertaining reactivity to MARV epitopes that are known to be protective in BALB/c mouse model prior to challenge with our novel mouse-adapted MARV-Ravn [17].

## Materials and methods

### Infection of BALB/c mice with nonlethal or lethal MARV virus

Six-week-old BALB/c mice obtained from Charles River (Wilmington, MA) were injected intraperitonealy with ~1000 plaque-forming units (PFU) of a nonlethal MARV Ravn isolate. The virus had been blind-passaged 17–20 times from mouse liver homogenates and did not produce clinical signs of disease when inoculated into naïve BALB/c mice. Mice were monitored for approximately 14 days before euthanasia and splenectomy. For lethal challenges, we used a later passage of lethal, mouse-adapted MARV Ravn that caused death 7–10 days after infection. Mouse adaptation was accomplished by serially passaging virus through the livers of SCID mice [34] and then BALB/c mice to obtain a lethal, mouse-adapted virus. The lethal, mouse-adapted MARV Ravn isolate was purified by plaque selection and then selected for its virulence towards BALB/c and C57BL/6 mice [17]. The pathogenesis of the mouse-adapted MARV Ravn was similar to the pathogenesis of guinea pig and nonhuman primate models with high viral titers in the blood, liver, lymphoid, and other organs; alterations in blood chemistries including markers of liver and kidney function; as well as loss of platelets and lymphocytes in the circulation [17].

### Stimulation of MARV-specific splenocytes with peptide pools, single 15 mers, and single 9 mers

Splenectomies were performed 14 days after infection with nonlethal MARV [17]. Spleens from all mice were pooled, homogenized, and washed through a 50-μm nylon filter. Cells were incubated in 0.144 M ammonium chloride lysis buffer to remove residual red blood cells. After a final wash in PBS, splenocytes were resuspended in RPMI/EHAA (Invitrogen, Carlsbad, CA) supplemented with 10% fetal bovine serum (FBS) (Hyclone Labs, Logan, UT), 2 μl of β-ME/500 ml (Sigma, St. Louis, MO), 10 mg/ml of brefeldin A, and 1 unit/ml of rhIL-2 in a 96-well U-bottom plate. Fifteen-mer and 9-mer MARV peptide sets were synthesized by Mimotope (Clayton, Victoria, Australia) and maintained in dimethylsulfoxide. The collection of all 15-mer peptides represented the entire translated GP, NP, and VP40 proteins. Nine-mer peptides were selected from each 15-mer peptide using HLA binding predictions for H2^*d *^developed by Parker *et al*. [35]. One μg of either overlapping 15-mer peptide pools, individual 15-mer peptides, or individual 9-mer peptides were added to each well containing 1 × 10^6 ^splenocytes and incubated for 5 h at 37°C in 5% CO_2_. An EBOV 15-mer peptide designated NP12 with no sequence homology to MARV NP peptides was used as a negative control. Positive controls included splenocytes stimulated with 100 ng of PMA and 1 μg of ionomycin.

### Analysis of splenocytes by flow cytometry

Stimulated splenocytes were centrifuged at 300 × g for 5 min, and cell pellets were resuspended in FACS buffer (PBS supplemented with 1% FBS, 0.1% sodium azide, and 10 mg/ml brefeldin A) containing either mouse anti-CD44-FITC or CD8-PerCP (BD Biosciences, San Jose, CA) diluted 1:100 and incubated for 30 min at 4°C. Washed splenocytes were fixed with buffered 1% paraformaldehyde and incubated for 15 min. Splenocytes were permeablized by adding FACS buffer and 0.5% saponin (permeablization buffer). Anti-mouse IFNγ (BD Biosciences, San Jose, CA) diluted 1:50 in permeablization buffer was added and incubated for 30 min. Splenocytes were fixed in 10% neutral buffered formalin and analyzed on a BD FACSCalibur system (BD Biosciences, Franklin Lakes, NJ). At the time of acquisition, the signal from EBOV NP12 (AEQGLIQYPTAWQSV)-stimulated splenocytes was used to determine the level of background in the experiment. A total of 10,000 splenocytes were counted. On average, 8–12% were CD8+ T cells that were gated to discriminate between levels of CD44 expression and IFNγ producing cells.

### CTL assays for prediction of lytic epitopes

PB-1 target cells were pulsed with 2 μg/ml of 9-mer peptides and incubated for 24 h. On the day of the assay, pulsed PB-1 target cells were labeled with ^51^Cr for 1 h. Effector cells were obtained from BALB/c mice 7 days after boosting with MARV VLPs. Briefly, mice were vaccinated with 100 μg of MARV VLPs (2 μg of QS21 per mouse) and boosted with 100 μg of MARV VLPs (2 μg of QS21) 2 weeks later. Effector cells, which were obtained 7 days after the MARV VLP boost, were added to the labeled, peptide-pulsed target PB-1 target cells and incubated for 4 h. Fifty μl of supernatant was transferred onto a filtered luma plate for analysis on a gamma counter.

### Adoptive transfer of stimulated splenocytes into BALB/c mice

Splenocytes removed from mice infected with nonlethal MARV were restimulated with 2 μg/ml of 9-mer peptide and incubated for 7 days at 37°C in 5% CO_2_. Recombinant human interleukin (IL)-2 and supernatant from concanavalin A-stimulated cells was added to the medium (EHAA/RPMI) on day 2. On day 7, stimulated splenocytes were purified using ficoll. Approximately 5 × 10^6 ^9-mer-stimulated splenocytes were transferred to each mouse in designated groups. As a control, some mice were given the same number of saline-stimulated splenocytes from mice previously infected with nonlethal MARV. Cell transfer preceded infection by approximately 3 h where each mouse was infected with 1000 PFU of lethal mouse-adapted MARV or EBOV [17,36]. Research was conducted in compliance with the Animal Welfare Act, federal statutes and regulations relating to animals and experiments involving animals, and adhered to principles stated in the Guide for the Care and Use of Laboratory Animals (National Research Council, 1996). The facility where this research was conducted was fully accredited by the Association for Assessment and Accreditation of Laboratory Animal Care International.

### Statistical analysis

Data collected from the animal survival studies were displayed on a Kaplan-Meyer plot. Statistical significance was determined by comparing each experimental group to the negative control group. Significance, when compared to the negative control group, was determined using log-rank tests with stepdown Bonferroni adjustment. All groups whose difference fell above the 95% confidence interval were considered significant. All statistical analysis was done using SAS software (SAS Institute Inc Cary, NC).

## Competing interests

The authors declare that they have no competing interests.

## Authors' contributions

WVK was responsible for planning and conducting experiments, data analysis, and manuscript preparation. KLW developed the mouse MARV model used in this study reviewed data, suggested experimental design, and provided pertinent topics of discussion that impacted the compilation of this manuscript. GGO provided new reagents, guidance for epitope analysis, and assay protocols that were essential in the completion of this research study. SB designed research provided project guidance, reviewed data, suggested experimental design, and reviewed data analysis and interpretations.
